# Double Deprotonation of CH_3_CN by an Iron‐Aluminium Complex**

**DOI:** 10.1002/anie.202219212

**Published:** 2023-03-09

**Authors:** Benedek Stadler, Nikolaus Gorgas, Andrew J. P. White, Mark R. Crimmin

**Affiliations:** ^1^ Department of Chemistry Imperial College London White City London W12 0BZ UK; ^2^ Institute of Applied Synthetic Chemistry Vienna University of Technology Getreidemarkt 9 1060 Vienna Austria

**Keywords:** Aluminium, C−H Activation, Dianions, Iron, Nitriles

## Abstract

Herein we present the first double deprotonation of acetonitrile (CH_3_CN) using two equivalents of a bimetallic iron‐aluminium complex. The products of this reaction contain an exceeding simple yet rare [CHCN]^2−^ dianion moiety that bridges two metal fragments. DFT calculations suggest that the bonding to the metal centres occurs through heavily polarised covalent interactions. Mechanistic studies reveal the intermediacy of a monomeric [CH_2_CN]^−^ complex, which has been characterised in situ. Our findings provide an important example in which a bimetallic metal complex achieves a new type of reactivity not previously encountered with monometallic counterparts.^[1, 2]^ The isolation of a [CHCN]^2−^ dianion through simple deprotonation of CH_3_CN also offers the possibility of establishing a broader chemistry of this motif.

## Graphical abstract

The double deprotonation of CH_3_CN was achieved using an iron‐aluminium complex, yielding products containing a simple yet rare [HC=C=N]^2−^ dianion, characterised by X‐ray crystallography. DFT calculations suggest that the mechanism proceeds via a stepwise double deprotonation of the substrate by two equivalents of the bimetallic complex, with the intermediacy of a [NCCH_2_]^−^ anion, which was observed spectroscopically in situ.

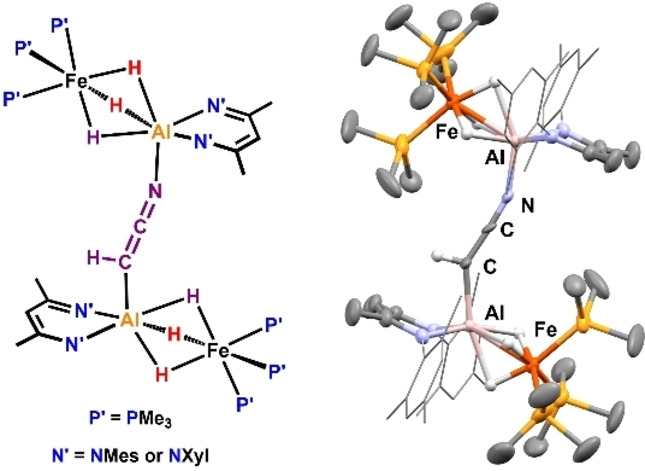

α‐Nitrile anions date back to the late 1800s. The deprotonation of CH_3_CN, by reaction with Na, was first reported by Holtzwart in 1889.^[3]^ In the 1960s, methods were developed for the isolation of NaCH_2_CN^[4]^ and LiCH_2_CN.[^5^, ^6^, ^7^] Tae and co‐workers recently reviewed the use of these anions in organic synthesis.^[8]^ Due to the limited delocalisation of the negative charge into the C≡N π‐system, they exhibit strong nucleophilic character and can be used to effect the cyanomethylation of numerous electrophiles.[^9^, ^10^, ^11^]

Despite widespread reports of molecular complexes containing the [CH(R)CN]^−^ anion,[^12^, ^13^, ^14^, ^15^, ^16^] examples which contain the [CRCN]^2−^ dianion are incredibly rare.[^17^, ^18^, ^19^, ^20^] The [CHCN]^2−^ dianion represents one of the simplest chemical building blocks available, this fragment contains only four atoms and 16 valence electrons. There are unanswered fundamental questions about the electronic structure of [CHCN]^2−^ and its binding modes to metal centres; theoretically, several structural motifs can be considered (Figure 1). These include 1,1‐dianion (**I**), 1,3‐dianion (**II**) and metallocarbene (**III**) species. To the best of our knowledge, only two structurally characterised compounds have been reported that contain the [CHCN]^2−^ motif; a diiron complex^[19]^ with connectivity reminiscent of **I** and tungsten cluster^[20]^ that appears to contain four units of **III**. A third, closely related species, Li_2_[(Me_3_Si)CCN] was reported in the late 1980s,^[17]^ as well as [Mes_2_Ge(C(Ph)CN)]_2_ in the 1990s.^[18]^ In the solid‐state, Li_2_[(Me_3_Si)CCN] forms aggregates which incorporate 1,3‐dimetallated dianions (cf. **II**) with external O or N donors stabilising the Li ions, and [Mes_2_Ge(C(Ph)CN)]_2_ is dimeric.

**Figure 1:**
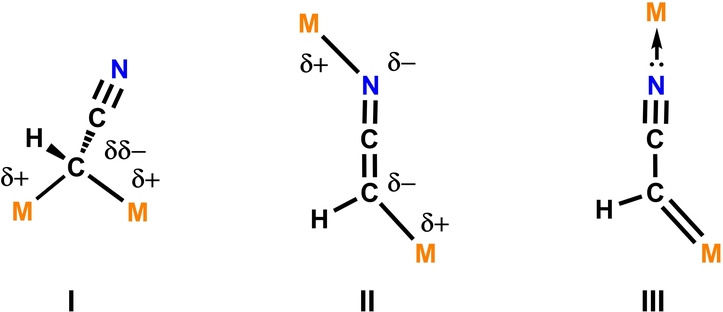
Theoretical structure types for [CHCN]^2−^ coordination to metals.

In this paper, we report the double deprotonation of CH_3_CN to form a [CHCN]^2−^ dianion. This was achieved by reaction of CH_3_CN with a highly basic Fe−Al bimetallic complex recently developed by our group.[^21^, ^22^] The resulting products contain two bimetallic metal fragments bridged by a 1,3‐dianion (structure type **II**). We analyse the electronic structure of the [CHCN]^2−^ dianion, the nature of its binding to the metals, and its mechanism of formation. Our findings provide new insight into the fundamental chemistry of an uncommon and understudied, yet extremely simple species, [CHCN]^2−^.

The reaction of **1 a** with acetonitrile (1 equiv.) in C_6_D_6_ at 25 °C proceeded with an immediate colour change from deep red to orange‐brown, corresponding to the formation of an intermediate (see below). Over the course of 3 days, **2 a** crystallised out of the reaction mixture in 33 % yield (Scheme 1). Single crystal X‐ray crystallographic analysis revealed that **2 a** is an unusual metal complex containing two Fe−Al units bridged by a *μ*_2_‐*κ*_C_‐*κ*_N_‐[CHCN]^2−^ dianion.^[23]^ Within this structure, the [CHCN]^2−^ dianion sits across a crystallographic centre of symmetry, meaning that the C, and N‐bound bimetallic sites cannot be unambiguously assigned. **2 a** is poorly soluble in common laboratory solvents precluding detailed solution‐state characterisation. Preparation of analogue addressed both these issues. Hence, reaction of **1 b** with CH_3_CN (0.5 equiv.) led to isolation of **2 b**, a direct analogue of **2 a** bearing 2,6‐xylyl groups in place of mesityl on the ligand periphery. **2 b** could be unambiguously characterised in both solution and solid state.

**Scheme 1:**
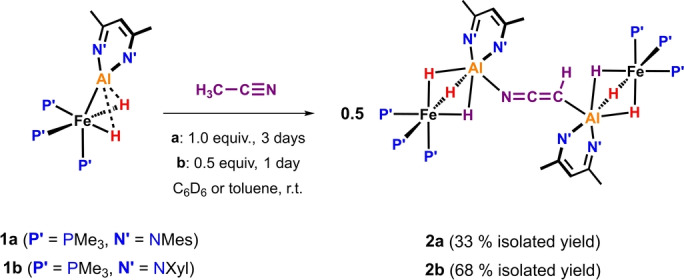
Synthesis of complexes **2 a**–**b**.

NMR analysis of **2 b** revealed a highly shielded resonance of δ_H_=0.56 ppm for the C *H* CN proton. **2 b** also featured two chemically inequivalent environments for the Al‐*μ*‐H_3_‐Fe hydrides, visible through two broadened resonances at δ_H_=−15.54 and −15.87 ppm, each integrating to 3H. Despite **2 b** showing good solubility in benzene, neither the ^13^C or ^14^N NMR resonances of the dianion fragment, nor any couplings in the respective 2D NMR spectra were observed. Variable temperature NMR experiments down to −50 °C in toluene‐[D_8_] yielded no further information. These observations are similar to those reported for Li_2_[(Me_3_Si)CCN], for which only the SiMe_3_ carbon resonance was reported.^[17]^ ^13^C isotopic labelling of the CH_3_CN fragment in **2 b‐[^13^C]** was achieved through reaction of **1 b** with ^13^CH_3_CN. **2 b‐[^13^C]** was characterised by a broad singlet at δ_C_=22.1 ppm (FWHM=51 Hz) assigned to the *C* HCN resonance broadened due to the adjacent quadrupolar S=5/2 ^27^Al nucleus. The ^13^C *H* CN resonance appeared as a doublet due to coupling to the S=1/2 ^13^C (^1^ *J*_H‐C_=127.4 Hz). IR spectroscopy of **2 b** revealed a strong band at 2038 cm^−1^ assigned as the C=N vibration. This is at a markedly lower wavenumber than the C≡N vibration in CH_3_CN (2251 cm^−1^). The experimental spectroscopic data is well reproduced by DFT calculations (Table S8).

The solid‐state structures of **2 a**–**b** are depicted in Figure 2. **2 a** crystallises in the *P* $\bar{1}$ , and **2 b** in the *P*2_1_/*n* space group, both with one solvent molecule in the asymmetric unit. Their structures are very similar, the main exception is the difference in the Fe−Al−Al−Fe torsion (**2 a**: 180.00 °; **2 b**: 68.57(9) °) which is likely caused by crystal packing effects. Both structures feature short C=C (**2 a**: 1.34(2) Å; **2 b**: 1.300(6) Å; CH_3_CN: 1.436(12) Å) and C=N (**2 a**: 1.16(2) Å; **2 b**: 1.206(6) Å; CH_3_CN: 1.149(12) Å) bond lengths clearly indicating a delocalised cumulene‐type structure of the [CHCN]^2−^ dianion.^[24]^

**Figure 2:**
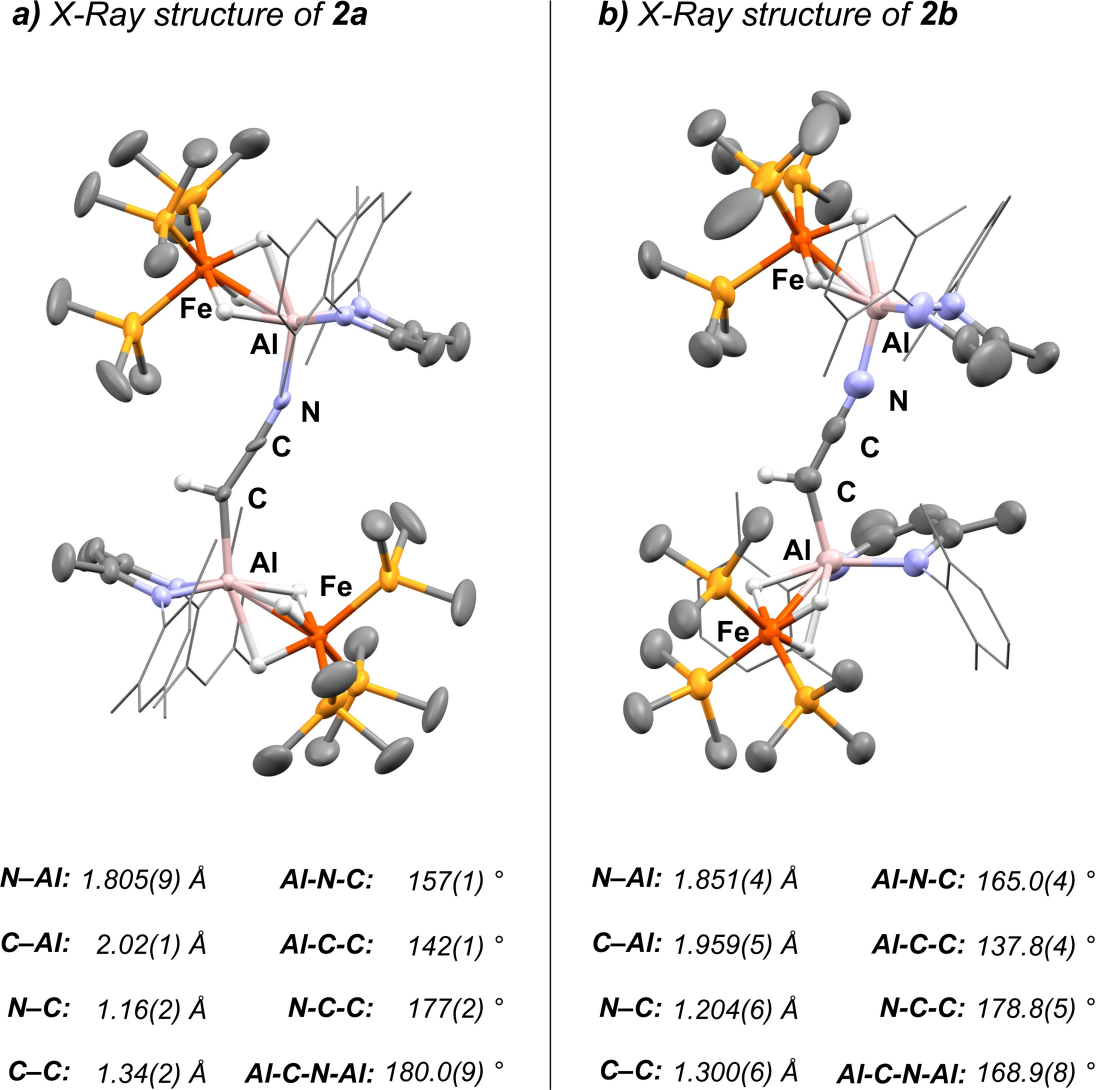
X‐ray structures of complexes **2 a**–**b**.

A Natural Bond Orbitals (NBO) analysis was carried out on **2 b** to gain insight into electronic structure of the [CHCN]^2−^ dianion (Figure 3).^[25]^ In **2 b**, the [CHCN]^2−^ dianion adopts a heterocumulene [HC=C=N]^2−^ motif. The [HC=C=N]^2−^ fragment is characterised by Wiberg Bond Indicies (WBIs) consistent with two conjugated double bonds (**2 b**: C=N 2.10, C=C 1.71).^[26]^ Using the same level of theory CH_3_CN shows discrete WBIs close to those expected for single and triple bonds (CH_3_CN: C≡N 2.91, C−C 1.08). Inspection of the NPA (Natural Population Analysis) charges shows near even charge localisation on the two terminal atoms of the [CHCN]^2−^ dianion (**2 b**: N −0.93, C −1.18, CH_3_CN: N −0.33, C −0.81).

**Figure 3:**
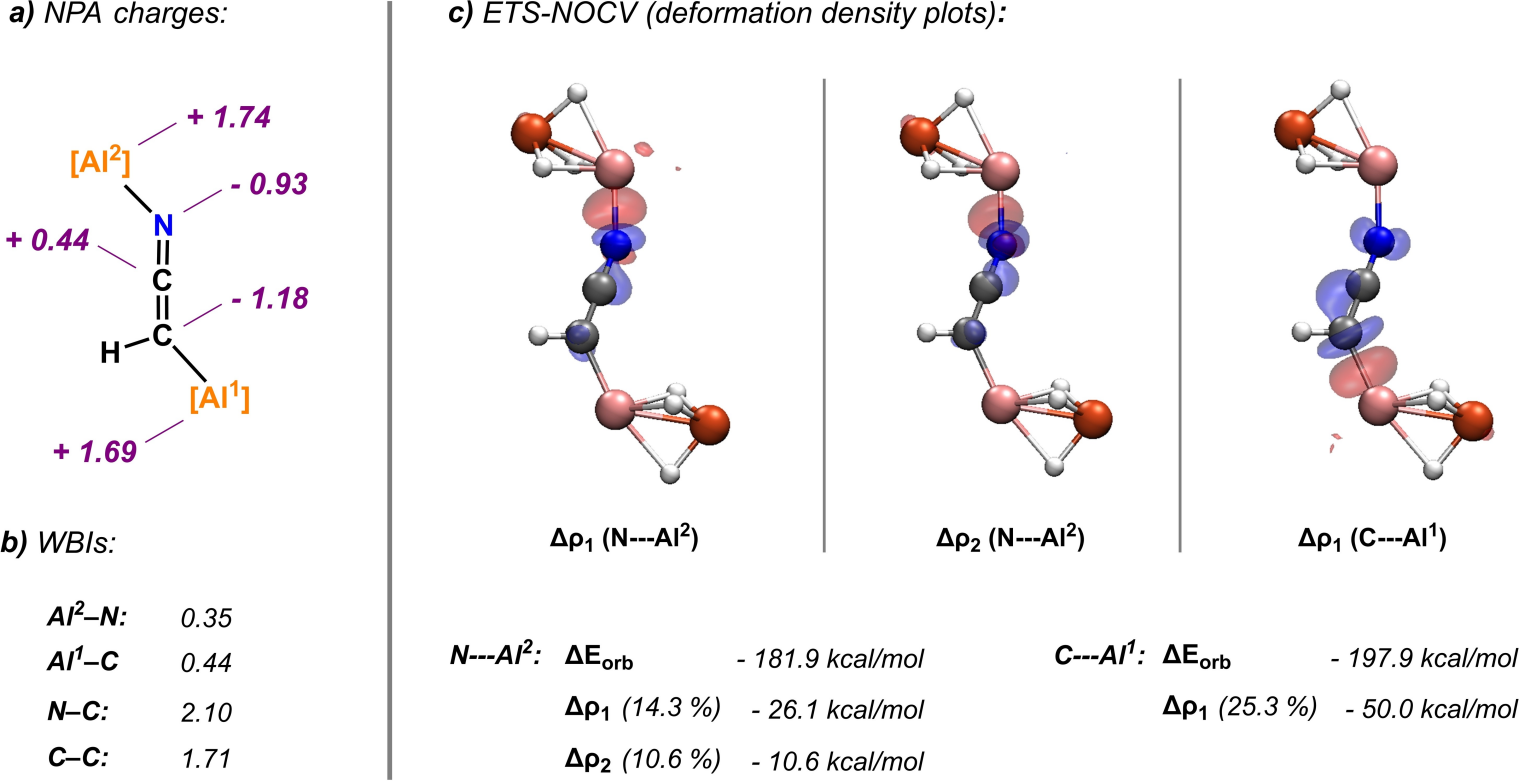
Bonding analysis of **2 b**. a) NPA charges and b) Wiberg bond indices from the NBO analysis; c) deformation density plots from ETS‐NOCV calculations (charge flow from blue to red).

NBO calculations also provide information on the nature of binding of the [CHCN]^2−^ dianion within **2 b**. The Al−C and Al−N WBIs are both lower than unity (**2 b**: Al^1^−C 0.44, Al^2^−N 0.35) and there is large positive charge localised on the Al nuclei (**2 b**: Al^1^ 1.69, Al^2^ 1.74). Second Order Perturbation theory reveals a series of donor‐acceptor interactions between the termini of the [CHCN]^2−^ dianion and the two Al centres (see Table S6 for details). These interactions were confirmed and quantified by ETS‐NOCV (Extended Transition State ‐ Natural Orbitals for Chemical Valence) calculations.^[27]^

Within the ETS‐NOCV approach the coordination modes were separated out, and binding of the N‐terminus and C‐terminus of the dianion considered independently. The total interaction energies were calculated to be Δ*E*_orb_(Al^2^−N)=−181.9 kcal mol^−1^ and Δ*E*_orb_(Al^1^−C)=−197.9 kcal mol^−1^ respectively. In both cases the most prominent contributions to the orbital interaction were identified as donation of electron density from sp^2^ hybrid orbitals of the terminal C/N atom of the dianion fragment into the Al p_z_ orbitals. Specifically, for the N−Al^2^ interaction: Δρ_1_ (N sp^2^_z_→Al^2^ p_z_)=−26.1 kcal mol^−1^, Δρ_2_ (N sp^2^_x_→Al^2^ p_y_)=−10.6 kcal mol^−1^ and for the C−Al^1^ interaction: Δρ_1_ (C sp^2^_z_→Al^1^ p_z_).=−50.0 kcal mol^−1^ (see Table S7 for details on ETS‐NOCV/fragmented NBO analysis of the bonding).

In combination the calculations suggest that **2 b** features a [CHCN]^2−^ dianion fragment possessing a C=C=N cumulene structure, which is stabilised through polarised covalent bonding to the two Al centres.

DFT calculations were also undertaken on the mechanism of the reaction of CH_3_CN with **1 b**.^[21]^ The computed pathway supports a stepwise double deprotonation of CH_3_CN by two equivalents of **1 b** (Figure 4). Initial, endergonic (Δ*G*=6.5 kcal mol^−1^) coordination of CH_3_CN to one molecule of **1 b** yields an unstable intermediate **INT‐1**. **INT‐1** can undergo an intramolecular deprotonation through **TS‐1**. In this transition state the substrate adopts a bent conformation. **TS‐1** leads directly to an Al ketene imide complex **3 b** containing the [CH_2_CN]^−^ anion. **3 b** is predicted to be experimentally accessible (see below). The first deprotonation step has a barrier of Δ*G* ^≠^=14.0 kcal mol^−1^ which is consistent with a reaction that is complete in <5 min at 25 °C. This activation barrier is comparable to that calculated for the C−H activation of pyridine by **1 a**, which goes through a very similar anchored transition state.^[21]^ Once formed, **3 b** can coordinate a second equivalent of **1 b** through the C‐terminus of the [CH_2_CN]^−^ anion to form **INT‐2** (Δ*G*=2.3 kcal mol^−1^) and effect a second deprotonation of the [CH_2_CN]^−^ moiety via **TS‐2** (Δ*G* ^≠^=10.3 kcal mol^−1^) generating **2 b** as the final reaction product. Both the first and second deprotonation steps involve early transition states and are highly exergonic (Δ*G*≈−30 kcal mol^−1^). An alternative pathway involving a direct deprotonation of CH_3_CN without pre‐coordination to Al has also been considered computationally but has significantly higher barriers and is unlikely to be operating (Figure S2).

**Figure 4:**
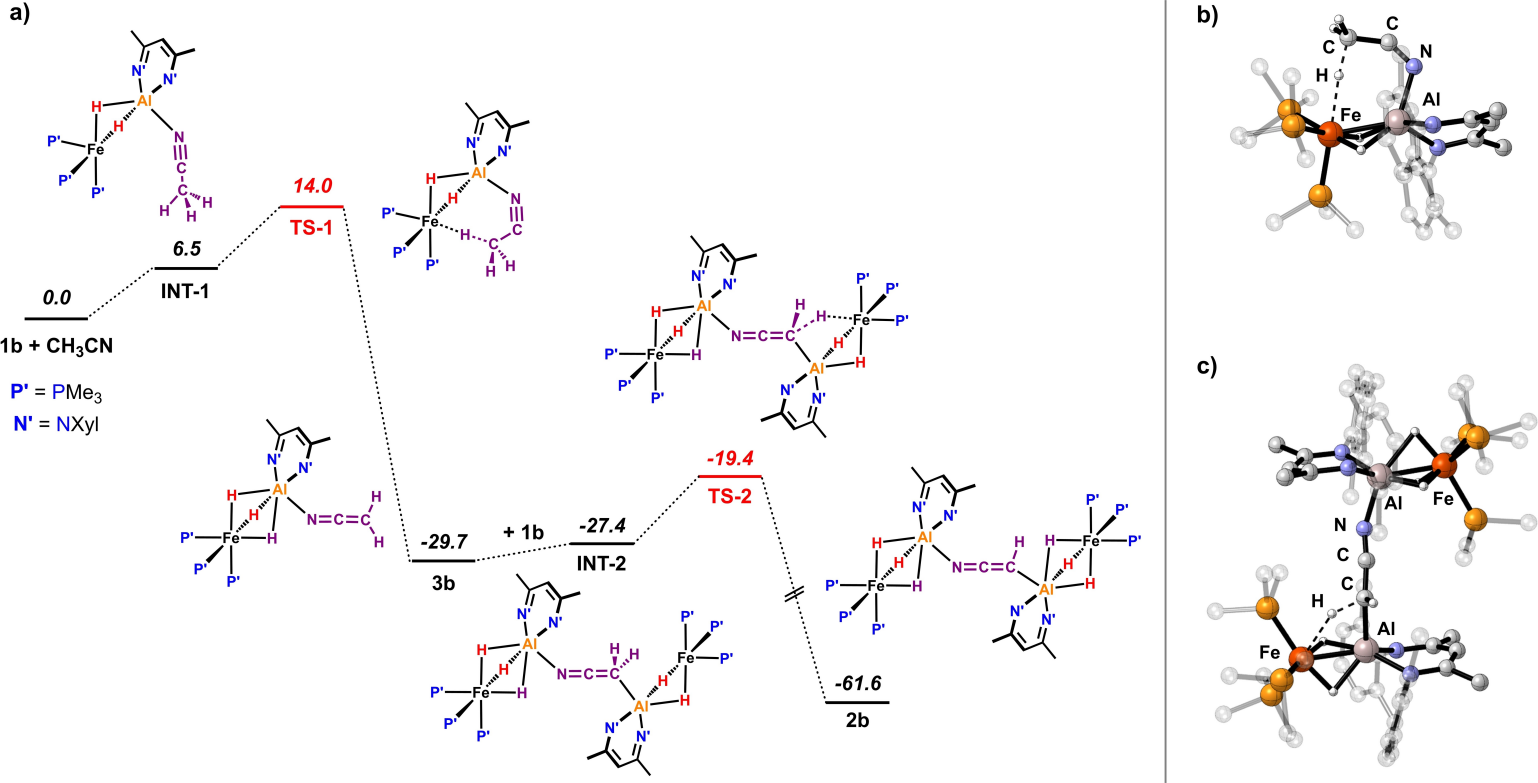
a) Calculated free energy profile for the double C−H activation of acetonitrile, Gibbs free energies in kcal mol^−1^; b) computed structure of **TS‐1**; c) computed structure of **TS‐2**.

NBO calculations provide further detail on the changes to electronic structure as CH_3_CN is doubly deprotonated (see Table S2 for the full NBO data along the reaction coordinate). The coordination of CH_3_CN to the Al centre of **1 b** subtly weakens the Fe−Al bond (Fe−Al WBI: **1 b**: 0.53, **INT‐1**: 0.44), increasing the charge localisation on Fe (**1 b**: −1.00, **INT‐1**: −1.12), and priming the complex for deprotonation. The heterocumulene structure of the substrate is essentially established by the first deprotonation step, with **3 b** showing similar WBIs (**3 b**: C=N 2.23, C=C 1.64) to **2 b**. Across the pathway from CH_3_CN→**INT‐1**→**TS‐1**→**3 b**→**INT‐2**→**TS‐2**→**2 b** there is an increase in the charge density concentrated on the [CH_*n*_CN]^3−*n*^ fragment (*n*=1–3); the sum of NPA charges on the C and N atoms trend −1.14→−1.24→−1.53→−1.67→−1.77→−1.99→−2.12.

Following the reaction between **1 b** and 9 equiv. of CH_3_CN by NMR spectroscopy in C_6_D_6_ revealed 94 % conversion to the postulated intermediate **3 b** in <5 min at 25 °C (Scheme 2). **3 b** shows a broadened singlet hydride resonance at δ_H_=−16.00 ppm, and a singlet ^31^P signal at δ_P_=−28.8 ppm. This latter resonance is characteristic for Al‐*μ*‐H_3_‐Fe species derived from proton transfer reactions.[^21^, ^22^]

**Scheme 2:**
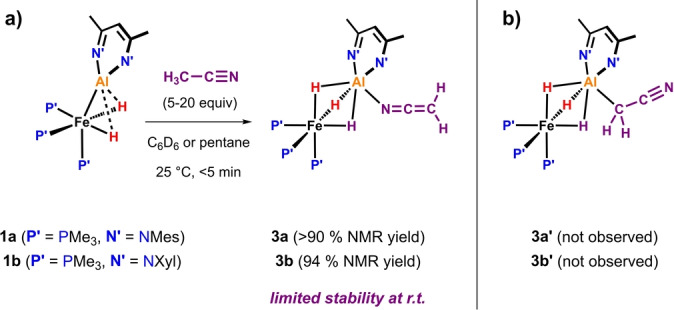
a) Experimental observation complex **3 b** through in situ NMR studies. b) Line drawing of the experimentally unobserved isomer of the intermediate, **3 b′**.

The C *H*_2_CN ^1^H NMR signal integrating to 2H can be found at δ_H_=1.81 ppm which is downfield of the corresponding resonance for C *H*_3_CN (δ_H_=0.58 ppm in C_6_D_6_).^[28]^ This resonance appears as a doublet with ^1^ *J*_C‐H_=166.6 Hz in **3 b‐[^13^C]**, the ^13^C labelled analogue of **3 b**. IR spectroscopic analysis supports the assignment of the intermediate as an N‐coordinated (**3 b**) rather than C‐coordinated (**3 b′**) isomer: **3 b** shows a strong band at 2067 cm^−1^ (DFT: 2076 cm^−1^) assigned as the C=N vibration. In contrast, DFT calculations put the C≡N vibration in **3 b′** at 2216 cm^−1^. DEPT‐135 as well as ^13^C{^1^H} NMR measurements also support this assignment (δ_C_(*C* H_2_CN)=3.8 ppm, DFT: **3 b**: 7.9 ppm cf. for **3 b′**: −0.9 ppm).

In conclusion, we present the first double deprotonation of CH_3_CN, achieved by reaction with two equivalents of a bimetallic Fe−Al complex. The products of deprotonation contain a coordinated [CHCN]^2−^ dianion. DFT calculations support charge localisation on the termini of the [CHCN]^2−^ dianion and its formulation as a [HC=C=N]^2−^ cumulene structure bound to the aluminium centres through polarised covalent interactions. Mechanistic studies suggest that the product is formed via a [CH_2_CN]^−^ monoanion intermediate. This species could be spectroscopically characterised *in situ*. DFT calculations suggest that the unique reactivity can be conceptualised in terms of two successive deprotonation steps, both of which are driven by the high basicity and unusual properties of the Fe−Al bimetallic complex. Our findings open up the possibility of a wider chemistry of simple [CHCN]^2−^ dianions including potential applications as chemical building blocks for synthesis.

## Conflict of interest

The authors declare no conflict of interest.

## Supporting information

As a service to our authors and readers, this journal provides supporting information supplied by the authors. Such materials are peer reviewed and may be re‐organized for online delivery, but are not copy‐edited or typeset. Technical support issues arising from supporting information (other than missing files) should be addressed to the authors.

Supporting Information

Supporting Information

Supporting Information

Supporting Information

## Data Availability

The data that support the findings of this study are available in the supplementary material (ESI) of this article available at https://doi.org/10.1002/anie.202219212, which contains the experimental procedures, details of calculations and characterization data (PDF), coordinates for DFT calculations (xyz), crystallographic data for **2 a** and **2 b** (cif, CCDC 2224389–2224390). Raw NMR, IR and computational data is available at the following repository: https://doi.org/10.14469/hpc/12264.
