# Supplementary material for: Double Deprotonation of CH3CN by an Iron‐Aluminium Complex
Source: Angew Chem Int Ed Engl. 2023 Mar 9;62(16):e202219212. doi: 10.1002/anie.202219212 (PMC10946928; doi:10.1002/anie.202219212)
Supplement: Supplementary file 2 — Supporting Information [file ANIE-62-0-s001.pdf]

checkCIF (basic structural check) running

Checking for embedded fcf data in CIF ...
Found embedded fcf data in CIF. Extracting fcf data from uploaded CIF, please wait . . . .

checkCIF/PLATON (basic structural check)

Structure factors have been supplied for datablock(s) 2a, 2b

THIS REPORT IS FOR GUIDANCE ONLY. IF USED AS PART OF A REVIEW PROCEDURE FOR PUBLICATION, IT SHOULD NOT REPLACE THE EXPERTISE OF AN EXPERIENCED CRYSTALLOGRAPHIC REFEREE.

No syntax errors found. CIF dictionary
Please wait while processing .... Interpreting this report

Structure factor report

Datablock: 2a

```
Bond precision:       C-C = 0.0035 A                Wavelength=0.71073
Cell:        a=11.8578(3)      b=13.2859(4)      c=14.2902(4)
             alpha=105.009(2)  beta=91.866(2)    gamma=113.041(2)
Temperature: 173 K
```

	Calculated	Reported
Volume	1978.18(10)	1978.18(9)
Space group	P -1	P -1
Hall group	-P 1	-P 1
Moiety formula	C66 H119 Al2 Fe2 N5 P6, C6 H6	C66 H119 Al2 Fe2 N5 P6, C6 H6
Sum formula	C72 H125 Al2 Fe2 N5 P6	C72 H125 Al2 Fe2 N5 P6
Mr	1412.25	1412.24
Dx,g cm-3	1.186	1.185
Z	1	1
Mu (mm-1)	0.551	0.551
F000	760.0	760.0
F000'	761.58	
h,k,lmax	16,17,19	15,17,18
Nref	10272	9070
Tmin,Tmax	0.803,0.927	0.852,0.943
Tmin'	0.803	

```
Correction method= # Reported T Limits: Tmin=0.852 Tmax=0.943
AbsCorr = ANALYTICAL
Data completeness= 0.883          Theta(max)= 28.767
R(reflections)= 0.0392( 7267)               wR2(reflections)= 0.1036( 9070)
S = 1.029               Npar= 435
```

The following ALERTS were generated. Each ALERT has the format
test-name_ALERT_alert-type_alert-level.
Click on the hyperlinks for more details of the test.

Alert level C

PLAT213_ALERT_2_C Atom C27 has ADP max/min Ratio ..... 3.4 prolat
PLAT220_ALERT_2_C NonSolvent Resd 1 C Ueq(max)/Ueq(min) Range 5.4 Ratio
PLAT222_ALERT_3_C NonSolvent Resd 1 H Uiso(max)/Uiso(min) Range 4.8 Ratio
PLAT242_ALERT_2_C Low 'MainMol' Ueq as Compared to Neighbors of P24 Check
And 2 other PLAT242 Alerts
Less ...

PLAT242_ALERT_2_C Low 'MainMol' Ueq as Compared to Neighbors of P28 Check
PLAT242_ALERT_2_C Low 'MainMol' Ueq as Compared to Neighbors of P32 Check

PLAT331_ALERT_2_C Small Aver Phenyl C-C Dist C51 --C53_b . 1.36 Ang.
PLAT910_ALERT_3_C Missing # of FCF Reflection(s) Below Theta(Min). 7 Note

Alert level G

PLAT154_ALERT_1_G The s.u.'s on the Cell Angles are Equal ..(Note) 0.002 Degree
PLAT300_ALERT_4_G Atom Site Occupancy of N40 Constrained at 0.5 Check
And 3 other PLAT300 Alerts
Less ...

PLAT300_ALERT_4_G Atom Site Occupancy of C41 Constrained at 0.5 Check
PLAT300_ALERT_4_G Atom Site Occupancy of C42 Constrained at 0.5 Check
PLAT300_ALERT_4_G Atom Site Occupancy of H42A Constrained at 0.5 Check

PLAT301_ALERT_3_G Main Residue Disorder ....(Resd 1) 4% Note
PLAT303_ALERT_2_G Full Occupancy Atom H1A with # Connections 2.00 Check
And 2 other PLAT303 Alerts
Less ...

PLAT303_ALERT_2_G Full Occupancy Atom H1B with # Connections 2.00 Check
PLAT303_ALERT_2_G Full Occupancy Atom H1C with # Connections 2.00 Check

PLAT380_ALERT_4_G Incorrectly? Oriented X(sp2)-Methyl Moiety ..... C14 Check
PLAT789_ALERT_4_G Atoms with Negative _atom_site_disorder_group # 4 Check
PLAT883_ALERT_1_G No Info/Value for _atom_sites_solution_primary . Please Do !
PLAT912_ALERT_4_G Missing # of FCF Reflections Above STh/L= 0.600 1159 Note
PLAT933_ALERT_2_G Number of HKL-OMIT Records in Embedded .res File 1 Note
PLAT978_ALERT_2_G Number C-C Bonds with Positive Residual Density. 3 Info
PLAT992_ALERT_5_G Repd & Actual _reflns_number_gt Values Differ by 2 Check

0 **ALERT level A** = Most likely a serious problem - resolve or explain
0 **ALERT level B** = A potentially serious problem, consider carefully
8 **ALERT level C** = Check. Ensure it is not caused by an omission or oversight
16 **ALERT level G** = General information/check it is not something unexpected

2 ALERT type 1 CIF construction/syntax error, inconsistent or missing data
11 ALERT type 2 Indicator that the structure model may be wrong or deficient
3 ALERT type 3 Indicator that the structure quality may be low
7 ALERT type 4 Improvement, methodology, query or suggestion
1 ALERT type 5 Informative message, check

Datablock: 2b

Bond precision: C-C = 0.0098 A Wavelength=1.54184

Cell: a=22.6316(6) b=11.7705(2) c=29.1563(7)
alpha=90 beta=93.302(2) gamma=90

Temperature: 173 K

	Calculated	Reported
Volume	7753.9(3)	7753.9(3)
Space group	P 21/n	P 1 21/n 1
Hall group	-P 2yn	-P 2yn
Moiety formula	C62 H111 Al2 Fe2 N5 P6, C7 H8	C62 H111 Al2 Fe2 N5 P6, C7 H8
Sum formula	C69 H119 Al2 Fe2 N5 P6	C69 H119 Al2 Fe2 N5 P6
Mr	1370.18	1370.16
Dx,g cm-3	1.174	1.174
Z	4	4
Mu (mm-1)	4.688	4.687
F000	2944.0	2944.0
F000'	2949.13	
h,k,lmax	28,14,36	27,14,36
Nref	15741	14854
Tmin,Tmax	0.510,0.928	0.601,0.928
Tmin'	0.463	

Correction method= # Reported T Limits: Tmin=0.601 Tmax=0.928
AbsCorr = ANALYTICAL

Data completeness= 0.944 Theta(max)= 73.969

R(reflections)= 0.0601(8470) wR2(reflections)= 0.1624(14854)

S = 1.009 Npar= 893

The following ALERTS were generated. Each ALERT has the format
test-name_ALERT_alert-type_alert-level.
Click on the hyperlinks for more details of the test.

Alert level C

PLAT213_ALERT_2_C Atom C61 has ADP max/min Ratio ..... 3.3 prolat
PLAT220_ALERT_2_C NonSolvent Resd 1 C Ueq(max)/Ueq(min) Range 3.6 Ratio
PLAT222_ALERT_3_C NonSolvent Resd 1 H Uiso(max)/Uiso(min) Range 10.0 Ratio
PLAT242_ALERT_2_C Low 'MainMol' Ueq as Compared to Neighbors of Fe2 Check
And 3 other PLAT242 Alerts
Less ...

PLAT242_ALERT_2_C Low 'MainMol' Ueq as Compared to Neighbors of P4 Check
PLAT242_ALERT_2_C Low 'MainMol' Ueq as Compared to Neighbors of P5 Check
PLAT242_ALERT_2_C Low 'MainMol' Ueq as Compared to Neighbors of P6 Check

PLAT245_ALERT_2_C U(iso) H3 Smaller than U(eq) Al1 by 0.018 Ang**2
PLAT245_ALERT_2_C U(iso) H6 Smaller than U(eq) Al2 by 0.020 Ang**2
PLAT331_ALERT_2_C Small Aver Phenyl C-C Dist C63 --C68 . 1.37 Ang.
PLAT331_ALERT_2_C Small Aver Phenyl C-C Dist C63A --C68A . 1.36 Ang.
PLAT341_ALERT_3_C Low Bond Precision on C-C Bonds .... 0.00976 Ang.
PLAT370_ALERT_2_C Short C(sp2)-C(sp1) Bond C1 - C2 . 1.30 Ang.
PLAT413_ALERT_2_C Short Inter XH3 .. XHn Hm ..Hl . 2.05 Ang.
1/2-x,-1/2+y,1/2-z = 2_545 Check
PLAT911_ALERT_3_C Missing FCF Refl Between Thmin & STh/L= 0.600 213 Report

Alert level G

PLAT002_ALERT_2_G Number of Distance or Angle Restraints on AtSite 39 Note
PLAT003_ALERT_2_G Number of Uiso or Uij Restrained non-H Atoms ... 31 Report
PLAT174_ALERT_4_G The CIF-Embedded .res File Contains FLAT Records 2 Report
PLAT176_ALERT_4_G The CIF-Embedded .res File Contains SADI Records 39 Report
PLAT177_ALERT_4_G The CIF-Embedded .res File Contains DELU Records 5 Report
PLAT178_ALERT_4_G The CIF-Embedded .res File Contains SIMU Records 4 Report
PLAT187_ALERT_4_G The CIF-Embedded .res File Contains RIGU Records 1 Report
PLAT188_ALERT_3_G A Non-default SIMU Restraint Value has been used 0.0200 Report
And 3 other PLAT188 Alerts
Less ...

PLAT188_ALERT_3_G A Non-default SIMU Restraint Value has been used 0.0200 Report
PLAT188_ALERT_3_G A Non-default SIMU Restraint Value has been used 0.0200 Report
PLAT188_ALERT_3_G A Non-default SIMU Restraint Value has been used 0.0200 Report

PLAT191_ALERT_3_G A Non-default SADI Restraint Value has been used 0.0400 Report
And 19 other PLAT191 Alerts
Less ...

PLAT191_ALERT_3_G A Non-default SADI Restraint Value has been used 0.0400 Report
PLAT191_ALERT_3_G A Non-default SADI Restraint Value has been used 0.0400 Report
PLAT191_ALERT_3_G A Non-default SADI Restraint Value has been used 0.0400 Report
PLAT191_ALERT_3_G A Non-default SADI Restraint Value has been used 0.0400 Report
PLAT191_ALERT_3_G A Non-default SADI Restraint Value has been used 0.0400 Report
PLAT191_ALERT_3_G A Non-default SADI Restraint Value has been used 0.0400 Report
PLAT191_ALERT_3_G A Non-default SADI Restraint Value has been used 0.0400 Report
PLAT191_ALERT_3_G A Non-default SADI Restraint Value has been used 0.0400 Report
PLAT191_ALERT_3_G A Non-default SADI Restraint Value has been used 0.0400 Report
PLAT191_ALERT_3_G A Non-default SADI Restraint Value has been used 0.0400 Report
PLAT191_ALERT_3_G A Non-default SADI Restraint Value has been used 0.0400 Report
PLAT191_ALERT_3_G A Non-default SADI Restraint Value has been used 0.0400 Report
PLAT191_ALERT_3_G A Non-default SADI Restraint Value has been used 0.0400 Report
PLAT191_ALERT_3_G A Non-default SADI Restraint Value has been used 0.0400 Report
PLAT191_ALERT_3_G A Non-default SADI Restraint Value has been used 0.0400 Report
PLAT191_ALERT_3_G A Non-default SADI Restraint Value has been used 0.0400 Report
PLAT191_ALERT_3_G A Non-default SADI Restraint Value has been used 0.0400 Report
PLAT191_ALERT_3_G A Non-default SADI Restraint Value has been used 0.0400 Report
PLAT191_ALERT_3_G A Non-default SADI Restraint Value has been used 0.0400 Report

PLAT192_ALERT_3_G A Non-default DELU Restraint Value for First Par 0.0000 Report
And 9 other PLAT192 Alerts
Less ...

PLAT192_ALERT_3_G A Non-default DELU Restraint Value for SecondPar 0.0000 Report
PLAT192_ALERT_3_G A Non-default DELU Restraint Value for First Par 0.0200 Report

PLAT192_ALERT_3_G A Non-default DELU Restraint Value for SecondPar 0.0200 Report
PLAT192_ALERT_3_G A Non-default DELU Restraint Value for First Par 0.0200 Report
PLAT192_ALERT_3_G A Non-default DELU Restraint Value for SecondPar 0.0200 Report
PLAT192_ALERT_3_G A Non-default DELU Restraint Value for First Par 0.0200 Report
PLAT192_ALERT_3_G A Non-default DELU Restraint Value for SecondPar 0.0200 Report
PLAT192_ALERT_3_G A Non-default DELU Restraint Value for First Par 0.0200 Report
PLAT192_ALERT_3_G A Non-default DELU Restraint Value for SecondPar 0.0200 Report

PLAT301_ALERT_3_G Main Residue Disorder ....(Resd 1) 14% Note
PLAT302_ALERT_4_G Anion/Solvent/Minor-Residue Disorder (Resd 2) 100% Note
PLAT302_ALERT_4_G Anion/Solvent/Minor-Residue Disorder (Resd 3) 100% Note
PLAT303_ALERT_2_G Full Occupancy Atom H2 with # Connections 2.00 Check
And 5 other PLAT303 Alerts
Less ...

PLAT303_ALERT_2_G Full Occupancy Atom H3 with # Connections 2.00 Check
PLAT303_ALERT_2_G Full Occupancy Atom H4 with # Connections 2.00 Check
PLAT303_ALERT_2_G Full Occupancy Atom H5 with # Connections 2.00 Check
PLAT303_ALERT_2_G Full Occupancy Atom H6 with # Connections 2.00 Check
PLAT303_ALERT_2_G Full Occupancy Atom H7 with # Connections 2.00 Check

PLAT304_ALERT_4_G Non-Integer Number of Atoms in ..... (Resd 2) 9.90 Check
PLAT304_ALERT_4_G Non-Integer Number of Atoms in ..... (Resd 3) 5.10 Check
PLAT412_ALERT_2_G Short Intra XH3 .. XHn H13 ..H7A . 1.88 Ang.
x,y,z = 1_555 Check
And 2 other PLAT412 Alerts
Less ...

PLAT412_ALERT_2_G Short Intra XH3 .. XHn H19 ..H1B . 2.06 Ang.
x,y,z = 1_555 Check
PLAT412_ALERT_2_G Short Intra XH3 .. XHn Ho ..H4C . 2.01 Ang.
x,y,z = 1_555 Check

PLAT720_ALERT_4_G Number of Unusual/Non-Standard Labels .... 90 Note
PLAT860_ALERT_3_G Number of Least-Squares Restraints .... 248 Note
PLAT910_ALERT_3_G Missing # of FCF Reflection(s) Below Theta(Min). 3 Note
PLAT912_ALERT_4_G Missing # of FCF Reflections Above STh/L= 0.600 659 Note
PLAT933_ALERT_2_G Number of HKL-OMIT Records in Embedded .res File 5 Note
PLAT941_ALERT_3_G Average HKL Measurement Multiplicity .... 1.7 Low
PLAT978_ALERT_2_G Number C-C Bonds with Positive Residual Density. 1 Info

---

0 **ALERT level A** = Most likely a serious problem - resolve or explain
0 **ALERT level B** = A potentially serious problem, consider carefully
15 **ALERT level C** = Check. Ensure it is not caused by an omission or oversight
62 **ALERT level G** = General information/check it is not something unexpected

0 ALERT type 1 CIF construction/syntax error, inconsistent or missing data
25 ALERT type 2 Indicator that the structure model may be wrong or deficient
41 ALERT type 3 Indicator that the structure quality may be low
11 ALERT type 4 Improvement, methodology, query or suggestion
0 ALERT type 5 Informative message, check

It is advisable to attempt to resolve as many as possible of the alerts in all categories. Often the minor alerts point to easily fixed oversights, errors and omissions in your CIF or refinement strategy, so attention to these fine details can be worthwhile. In order to resolve some of the more serious problems it may be necessary to carry out additional measurements or structure refinements. However, the purpose of your study may justify the reported deviations and the more serious of these should normally be commented upon in the discussion or experimental section of a paper or in the "special_details" fields of the CIF. checkCIF was carefully designed to identify outliers and unusual parameters, but every test has its limitations and alerts that are not important in a particular case may appear. Conversely, the absence of alerts does not guarantee there are no aspects of the results needing attention. It is up to the individual to critically assess their own results and, if necessary, seek expert advice.

Publication of your CIF in IUCr journals

A basic structural check has been run on your CIF. These basic checks will be run on all CIFs submitted for publication in IUCr journals (*Acta Crystallographica*, *Journal of Applied Crystallography*, *Journal of Synchrotron Radiation*); however, if you intend to submit to *Acta Crystallographica Section C* or *E* or *IUCrData*, you should make sure that full publication checks are run on the final version of your CIF prior to submission.

Publication of your CIF in other journals

Please refer to the *Notes for Authors* of the relevant journal for any special instructions relating to CIF submission.

PLATON version of 28/11/2022; check.def file version of 28/11/2022

Datablock 2a - ellipsoid plot

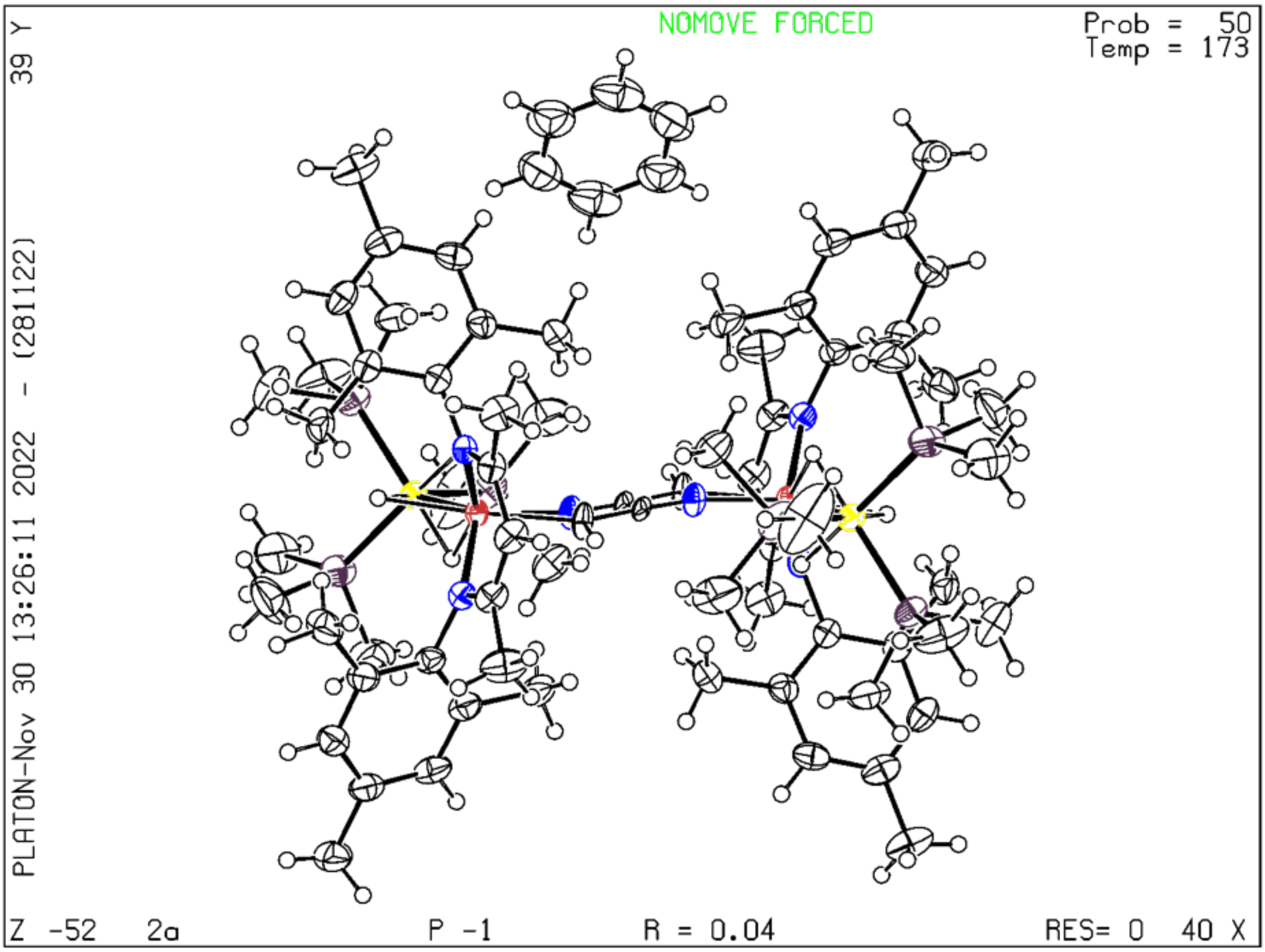

Datablock 2b - ellipsoid plot

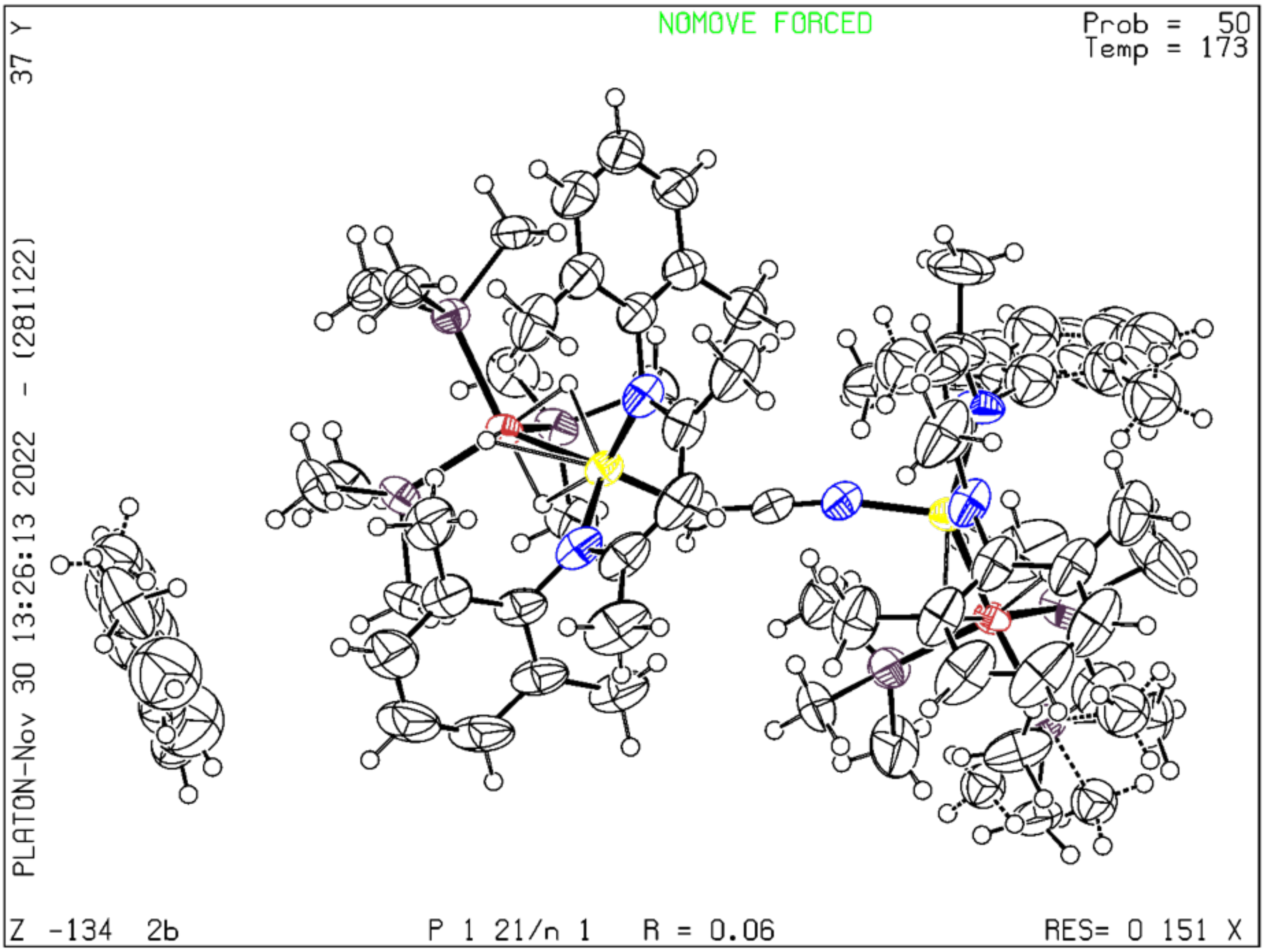

Download CIF editor (publCIF) from the IUCr
Download CIF editor (enCIFer) from the CCDC
Test a new CIF entry
